# Theoretical Basis for a Group Intervention Aimed at Preventing High School Dropout: The Case of ‘Guttas Campus’

**DOI:** 10.3390/ijerph192417025

**Published:** 2022-12-18

**Authors:** Gro Hilde Ramsdal, Rolf Wynn

**Affiliations:** 1 Department of Social Education, Faculty of Health Sciences, UiT The Arctic University of Norway, 9404 Harstad, Norway; 2 Department of Clinical Medicine, Faculty of Health Sciences, UiT The Arctic University of Norway, 9038 Tromsø, Norway

**Keywords:** school dropout, school re-enrolment, group intervention, theoretical basis, positive psychology, positive education, character strength

## Abstract

School dropout may have important negative consequences for the individual as well as for society. It is therefore important to help students stay in school. Group interventions have been developed to reduce dropout, but the theoretical underpinnings of such programs are not always obvious. This study focuses on the Norwegian dropout-prevention program named ‘Guttas Campus’ (The Boys’ Camp). We draw on published and unpublished research, other sources of written information, discussions with stakeholders, and direct observation of the intervention, to identify central theories that form the basis of the intervention. These theories are briefly presented, and the impact of the ideas on the intervention is analysed.

## 1. Introduction

School dropout represents a significant social problem within the OECD area due to its detrimental consequences [1]. Dropping out of upper secondary school is associated with unemployment, low wages, incarceration, drug abuse, long-term disability, and dependence on social security [2,3,4,5,6,7]. Thus, much effort has gone into developing interventions that can ease adolescents’ transition from secondary education into employment or higher education [8,9,10,11,12]. Nevertheless, the transition from lower to higher secondary schools seems to carry a particular risk of dropout. The lower the school engagement and the higher the absenteeism in tenth grade, the less likely Norwegian adolescents were to complete upper secondary education, after 5 years and after 14 years [13]. Markussen and Daus [13] conclude that absenteeism in lower secondary school is a warning sign of disengagement processes ending in high school dropout. Furthermore, studies show how the results of school disengagement processes depend heavily on grades from the last year in lower secondary school. When comparing students matched on other variables, students with good grades in tenth grade have an advantage that the other students never catch up with during higher secondary school [14]. Students with low grades and high absenteeism on the other hand, are often ignored in school because students with even higher risk of dropout, due to serious behavioral and academic problems, get more attention [1]. Left to care for themselves, the disengaged students often increase their risk of school dropout. Students that have reached this kind of tipping point can, with a relatively small effort, be identified and given sufficient support to tip them in the direction of school completion [1]. When teachers are supportive of students’ autonomy, this may also increase the students’ psychological well-being [15] and thereby further contribute to school completion.

During the period 2013–2019, 74% of the male students and 82% of the female students completed upper secondary school in Norway, indicating that male students seem to have more problems completing than female students. Falch, Borge, Jujala, Nyhus and Strøm [16] speculate that the greater part of this gender difference is related to boys having lower grades than girls in primary school. In a national report from 2010, more male students in lower secondary education reported themselves to be bored and to experience classes as too theoretical [17]. Students who report many conflicts at school are also less motivated and have lower grades, and most of them are boys.

Furthermore, dropping out of school means an increased risk of incarceration [2], and interventions increasing school completion should thus be effective in preventing crime. However, the effect is larger for boys because more of them become incarcerated in the first place [18]. Furthermore, school dropout implies a 21% increase in the risk of becoming permanently dependent on social security [3], and there is an increase in young men receiving disability benefits, particularly among men under 20 [19]. Thus, present research indicates that dropping out of school may be associated with particularly negative consequences for young men.

The Norwegian educational system is organized into three levels: 1. Elementary school (ages 6–13); 2. Lower secondary school (ages 13–16); and 3. Upper secondary school (ages 16–19) [20]. Elementary school and lower secondary education are mandatory, and the schools are mostly municipal. After the reform in 1994, upper secondary school has been a statutory right for all 15–16-year-olds. In Norway, 96–97% of every cohort enters this equivalent of high school after completing 10th grade [21]. These equivalents of high school are mainly public schools (in the American sense of the term) and are attended by 93% of the students [20]. The students can choose between a three-year program called general studies, qualifying them for higher education, or four-year vocational programmes, qualifying them for a wide range of occupations [21].

A number of school dropout prevention interventions have been introduced in Norway. One relatively new is called ‘Guttas Campus’ (GC). GC is a group intervention aimed at giving 9th grade boys with a high risk of dropping out of upper secondary school the academic and social support needed to transition successfully into upper secondary school and eventually complete upper secondary school and find employment.

The GC intervention was developed around the practical observations and academic understanding of a group of experienced educators and psychologists. They had spent decades interacting with children and adolescents in schools, kindergartens, home environments, and in therapy. They had experienced how many children were disappointed and frustrated when trying to fulfil the demands of the educational system while protecting their personal well-being. Therefore, they developed an educational intervention to inspire aspirations in young people at risk of school dropout and to teach them the necessary skills to fulfil these aspirations, so they can feel good about themselves. The Norwegian version described here was inspired by the Danish program called DrengeAkademiet [22]. The Norwegian program is designed as a two-week learning camp with follow-up and consists of three elements. First, the boys participate in a learning camp with explicit expectations, rules, and agreements for a group of 25–40 boys. Participants work systematically with reading, writing, and math. All learning activities have a specific structure and clear objectives and are individually customized. A main goal is learning to learn. The learning camp also focuses on positive habits like adequate sleep, healthy diet, physical activity, socializing, and very limited access to mobile phones. Through the whole program, there is an explicit focus on what they call character strengths as a way of learning to learn and cope with challenges. The staff are competent adults with a high degree of legitimacy both professionally and personally. They are chosen according to their ability to be role models the boys can identify with and respect. Second, the boys participate in mentor groups. After the learning camp, the boys are monitored in groups two times a month. The mentor centres are designed to support and motivate the participants to uphold the positive habits from the camp, help with homework, stimulate academic interest, and continue working with the character strengths and their social well-being. Third, the parents get a follow-up involving mandatory parent meetings before and after the learning camp.

This model has been evaluated in Norway, finding substantial academic improvement and low dropout rates during lower secondary school and during the first two years of higher secondary school [23]. In Denmark, studies have shown mixed results, but recent improvements in the DrengeAkademiet program seem to have led to more consistent positive outcomes, at least in the short term [22,24]. One of the originators of the Norwegian version, Tronsmo [25], pointed to the integration of educational, social, and relational factors and coherence as the strengths of this model, stating that the long-term effects are dependent on the success of the mentor group follow-up. Furthermore, testing the Danish version of the model, Rosholm and colleagues [22] found that compared to a control group, more of the learning camp participants completed lower secondary school.

While there has been some empirical research on the effects of GC, suggesting that this group intervention may contribute to the prevention of school dropout, there is a need to further explore the theoretical underpinnings of the program. Examining the theoretical basis of the program will help us gain more understanding of the pedagogical and psychological processes that are in effect in the program. This may be useful in the further improvement of the program and may also be relevant to researchers and other stakeholders designing group-based interventions for youth at risk of dropping out of school. Consequently, the purpose of this study is to examine the theoretical basis of the dropout-prevention program GC. We had the following research questions: (1) Which theories are central to the GC program?; (2) Which scientific literature underlies the theories? i.e., referred to in research question no. (1); and (3) How do these theories influence the GC program in practice?

## 2. Materials and Methods

This qualitative study aimed to examine the theoretical underpinnings of the GC program. We followed a four-phase research process.

In the first phase, we gathered data for the analysis from different sources, including written information, videos, informal meetings and discussions, and observations. The data were gathered in a three-step procedure: In the first step, we gathered all available written information, published and unpublished research, evaluation reports, briefs, publicly available videos from prior camps, information leaflets sent to participants and parents, etc., that included direct and indirect information about the theories and ideas that formed the basis of the program. The first author also attended a conference on dropout organized by GC. In the second step, the first author had several informal meetings with the program’s stakeholders, including its founders, its current management, and program instructors, and discussed central ideas and theories. In the third step, the first author observed preparations for the camp and parts of the camp itself as well as the graduation ceremony and made notes regarding possible theoretical influences.

In the second phase, following the gathering of these different sources of data, we examined all the data, identified and named ideas and theories that appeared central to the program, and created a list of relevant theories. Furthermore, we sought to identify the most important theories; those that were given more emphasis in the data and that occurred in different sources of data were considered to be more central. For instance, theories that were mentioned in published research as well as in informal discussions with stakeholders were understood to be more central to the GC program. Following this procedure, we were able to create a short list of the four theories we found to be most influential to the program.

In the third phase, we identified relevant literature underlying the four central theories and reviewed the literature with the purpose of giving a short description of the relevant theories, as presented in Section 3.1 of the Results section.

In the fourth phase, we analysed how these four core theories influenced the program in practice, by comparing the theories to the day-to-day teaching, routines, and events of GC. By following this procedure, we were able to substantiate our findings regarding which theories were most influential. Phase four resulted in a description of how the theoretical underpinnings of the GC program were implemented in actual practice in the program, as presented in Section 3.2 of the Results section.

## 3. Results

### 3.1. The Theoretical Basis for a Group Intervention

We identified four main theoretical underpinnings of the GC program: positive psychology, positive education, character strengths, and visible learning.

#### 3.1.1. Positive Psychology

A main inspirational source for GC is positive psychology. Psychology in general has successfully focused more on human shortcomings and illnesses than on virtues and achievable aspirations, according to Maslow [26], who first used the term “positive psychology”. After World War II, psychology was more about healing and repairing damage than about building positive qualities and well-being [27,28]. More than forty years later, Seligman re-introduced the term positive psychology, aiming to overarch and unite scattered lines of theory and research and to unite researchers focusing on human strengths, positive attributes, and what makes life worth living [29,30]. Building on pioneering work by Rogers, Maslow, Jahoda, Erikson, and Deci and Ryan, Seligman [31] describes how he realized that raising children is about more than fixing their problems; it is also about “identifying and nurturing their strongest qualities, what they own and are best at, and helping them find their niches in which they can live out these positive qualities”. In line with these thoughts, he asserts that concentrating on the positive qualities in people will act as a buffer against psychopathology and constitute an effective protection against various types of risks.

In line with this thinking, a teenager who is taught interpersonal skills and an optimistic attitude and who is helped to find flow experiences in sports is less at risk of mental health problems and substance abuse [32]. This means adhering to theories that view individuals not as passive pawns or respondents but as decision makers with preferences and possibilities of becoming self-regulating and masterful or, in negative circumstances, becoming helpless [31,33]. With its focus on optimal functioning, positive psychology can be understood as “a distinct way of viewing the human condition” [34]. It is probably this take on the human condition and the interest in people’s potential to thrive and grow, given the right context, that makes positive psychology so intriguing to educators. Many have been inspired in their effort to bring positive psychology into the educational system, thus forming a movement called “positive education”, a movement aspiring to combine academic skills and life skills to increase well-being and learning [35].

#### 3.1.2. Positive Education

The positive education movement advocates well-being programs in school because they believe that these programs, first, promote skills and strengths valued by most parents; second, improve well-being and behaviour; and finally, facilitate engagement in learning and achievement. According to Norrish, Williams, O’Conner, and Robinson [36], “positive education seeks to combine principles of Positive Psychology with best-practice teaching and with educational paradigms to promote optimal development and flourishing in the school setting”. The pathways for integrating positive psychology into school are associated with Seligman’s PERMA theory: focusing Pleasurable experiences, fostering Engagement in positive activities, enhancing Relationships, promoting Meaning and purpose, and supporting Accomplishments [37]. In his Geelong Grammar School (GGS) Model for positive education, Seligman also targets positive health [36].

Although the core message of positive psychology—to increase our attention to the relevance of positive emotional experiences in life and at school—does make immediate sense, this viewpoint has met with some valid criticism. According to Ciarrochi and colleagues [34], facing this criticism is vital if the positive education movement is to succeed in applying positive psychology in schools. Therefore, these researchers address some of the most important aspects of this criticism. First, focusing so strongly on the private experiences—how people feel and think—and how these experiences fuel activities aimed at good grades, wealth, health, and well-being may cause the positive education movement to underrate the influence of contextual factors. There is a risk that the positive education movement may reinforce the general tendency to over-emphasize personality-based explanations over the power of context, also called the fundamental attribution error [38]. Consequently, this line of thinking might enable politicians to place the responsibility for social problems and ill health with the individual.

Second, Ciarrochi and colleagues [34] state that there is also a risk of underestimating the relevance of negative affect by not dealing with it properly. Negative affect is a meaningful and inherent aspect of human lives, and overestimating the importance of feeling happy might therefore stimulate the avoidance of negative experiences. Moreover, in their review, Chawla and Ostafin [39] concluded that experiential avoidance was found to be a factor in the aetiology of maladaptive behaviour. Third, Ciarrochi and colleagues [34] point to the criticism concerning positive psychology’s focus on the pursuit of happiness and how this may set them up for disappointment because some periods in life may require other priorities; for example, when going through divorce, bereavement, or financial crisis. Understanding what makes oneself happy may also be challenging: intoxication may, for example, seem a good idea in the short run.

To remediate such problems, Ciarrochi and colleagues [34] propose context-focused positive psychology (CPP) interventions promoting “flexible, value-consistent behaviour”. Responding to the criticism described above, Ciarrochi and colleagues [34] propose the DNA-V model. The model is constructed to help young people face adversities and challenges with flexible responses while staying true to their values and thus building vitality and resilience. Values, here, refer to qualities of effort that are experienced as meaningful and important to the individual. Examples of such values include relating to others, challenging oneself and being curious to learn, engaging in physical activity, and caring for oneself. Thus, whether a behaviour “works” for a young person in a particular context is decided by whether it is useful in building value and vitality. This can only be determined by the young people themselves. According to Cohen and Sherman [40], clarification and affirmation of values are beneficial for one’s health, education, and relationships.

The DNA-V (Discoverer, Noticer, Advisor–Value) model consists of six main recommendations. First, the model recommends empowering contexts for young people to help them clarify their values and act on them [34]. Second, the model is focused on helping young people use their “advisor” or inner voice to navigate the world, so that they do not have to navigate by trial and error every time and so that they can improve their problem-solving skills through the increased use of language. The model is also intended to help young people clarify when listening to their inner voice or advisor is helpful in producing vitality and affirming values and when it is not. Third, the model concentrates on helping young people be aware of internal and external signals in their present context without pushing them away by, for example, distracting themselves with videogames or becoming overwhelmed and acting out in anger. The “noticer” function can help young people notice and name their feelings, thoughts, and physical reactions, and thus give them more time to find a way to regulate their emotions and their behaviour. Fourth, the model aims at developing young people’s inclination to be a “discoverer” by helping them explore new skills and resources that can broaden their context and help them adapt to the adult world they are entering. Again, the model is about helping them discover what kind of risk-taking can assist them in building value and vitality rather than avoiding risk in all contexts. Fifth, there is a focus on helping young people take a perspective on themselves in contrast to becoming one with their unhelpful self-views. By giving them simple framings like I–YOU, HERE–THERE and NOW–THEN, the model is providing more flexible and compassionate self-views, preventing unhelpful self-views like “I am a bad reader” from permeating their selves to become part of their essence; instead, the model helps them open up to new possibilities in self-views like “I have been a bad reader” (now–then). Accessing more flexible and compassionate self-views can then open up the possibility to increase their social skills and their ability to step into other people’s shoes. And finally, since group membership is important to young people, the model includes using prosocial groups to teach nurturing and cooperative behaviour [34].

Summing up the contextual, positive psychology perspective, Ciarrochi and colleagues [34] state that instead of seeing demotivated school students as broken and in need of fixing, they try to provide environments where these young people can thrive and focus on teaching them to change their context to provide more vitality and meaning.

#### 3.1.3. Character Strengths

One concept associated with positive psychology attracted particular attention from the three educators while developing the theoretical basis of the GC intervention, namely their second inspiration: character strengths. Positive psychology had provided an overall perspective on how to build and maintain positive educational aspirations, but the perspective needed a description of how to realize and put into action this overall positive psychology.

Context-focused psychology (CPP) concentrates, as described above, on the importance of context and situational factors in influencing youth behaviour and development [34]. This perspective was developed to take into consideration the valid criticism of positive psychology. That said, “everyone brings something to the situation”, according to Peterson and Seligman [41] and, they maintain, the most important thing is character, in the form of positive traits. Good character is defined as the something all parents look for in their children, teachers look for in their students, and friends in each other [42]. Peterson and Seligman [41] distinguish among virtues, character strengths, and situational themes in their classification. There are six classes of virtue—wisdom, courage, humanity, justice, temperance, and transcendence—that are made up of 24 character strengths. Virtues are the core characteristics defined as basic values in philosophy and religion and are declared to be universal. Furthermore, this theory speculates that when all these virtues occur and are above threshold for an individual, it means that the person is considered to be of good character.

The 24 character strengths are explained to be the processes or mechanisms that define the virtues: their trait-like manifestation [41]. The character strengths constitute the routes to displaying the virtue; for example, courage can be achieved through character strengths like using one’s willpower to accomplish goals in the face of resistance, persisting despite obstacles, not shrinking from a challenge, being genuine in one’s self-presentation, and acting sincerely but also approaching life with vitality and enthusiasm: going all in. All these components of good character are seen as core components of optimal youth development because they enable individuals to be at their best and manifest their potential [42]. While skills, abilities, and knowledge may be important, the lack of “good character” will include not having the courage to perform morally or socially valued tasks or not having the persistence to hold on to these values when doing so comes with a cost. This is one of the main explanations for why some consider it essential to bring good character interventions into the school arena: to promote the intellectual, social, emotional, and ethical development of young people [43]. In line with this thinking, the UN Convention on the Rights of the Child has chosen personal development and working towards fulfilment of one’s potential as the core obligations in education. Consequently, these dispositions to act, think, and feel in ways that promote youth development, resulting in positive consequences for the individual as well as for society, have long been regarded as a central aspect of human development [44].

Nevertheless, 24 character strengths are a lot to work with when thinking in terms of interventions to improve youth development, school achievements, or adjustment. Thus, there was a call for a more manageable list of strengths [45]. There have been many answers to this call. Lickona and Davidson [46] came up with two distinct but related factors called performance character and moral character. Performance character implies qualities needed to obtain excellence, such as perseverance and self-control, while moral character implies qualities needed to succeed in personal relationships and moral behaviour, like integrity, caring, and respect. A third dimension of character was suggested by Baehr [47] and Ritchhart [48], namely intellectual character, including traits like curiosity and open-mindedness. This character strength is not the same as cognitive ability, because a person can be intelligent and knowledgeable and still be arrogant, superficial, or close-minded and not have what Baehr [49] describes as a disposition for lifelong learning. Furthermore, such intellectual character skills are important because they have been found to rival the predictive power of pure cognitive skills.

The National Research Council (NRC) contributed to this search for a manageable list of character strengths by going through a large research database seeking to identify dimensions of “21st century skills”. In its final report, the NRC names three “competency clusters” that have increasing significance in modern society [50]. First, there are “interpersonal competencies” dealing with people’s ability to cooperate, work together, and get along. Second, there are “intrapersonal competencies”, including traits like self-control and grit; and finally, there are “cognitive competencies” like reasoning and critical thinking. The NRC recommended the development of all three competencies in schools. However, this tripartite model lacked empirical validation. According to Park et al. [44], most empirical research on the organization of character in school-aged children has used the Values in Action Youth Inventory (VIA-Y) [42]. Studies investigating the factor structure of the VIA-Y, although showing conflicting results, have indicated four or five factors, and all suggested models have included the factors of the competency clusters of the ”21st century skills:” interpersonal, intrapersonal, and cognitive competencies.

Trying to answer the question of which competencies, other than cognitive competencies, to promote in school children, Park and colleagues [44] set out to investigate the factor structure of character. Using exploratory factor analysis, they found a three-factor structure consisting of, first, interpersonal factors of character, including interpersonal self-control, gratitude, and social intelligence; second, intellectual factors of character, including zest and curiosity; and finally, intrapersonal factors of character, including academic self-control and grit. Interpersonal character was found to predict positive peer relationships, while intellectual character predicted class participation and intrapersonal character predicted grades [44]. Consequently, there seems to be a relatively broad consensus about the relevance of this tripartite model as a basis for school intervention programs.

#### 3.1.4. Visible Learning

Finally, there was a fourth inspiration for the development of the GC intervention: visible learning. Visible learning is the name of a book by John Hattie [51], in which he reassesses the research on the conditions of successful school learning. The reassessment is based on 800 meta-analyses and 52,637 individual studies, resulting in the emergence of 138 factors that influence successful school learning. Hattie [51] arranged the factors into six thematic groups: displaying the contributions to school learning from the student, the home, the school, the teacher, the curricula, and from teaching approaches. Results showed that, of the six thematic groups, the teacher showed the strongest influence on successful school learning. The overall message is that teaching must be visible to the student and that the resulting learning must be visible to the teacher. Thus, seeing becomes essential to learning, in the sense that taking a certain perspective is essential [52]. The teacher must see the learning through the eyes of the pupil, and the pupils must gradually see themselves as their own teacher. Hattie describes the good teacher as active, responsible, directive, and cautious [52]. The teaching must be visible to the student, and thus the teacher becomes directive; however, the teacher must also cautiously listen to and watch the student’s response. Through this kind of seeing, the teacher is able to scaffold learning and develop meta-strategies for learning. The ideal teacher is well aware of the learning abilities and the proximal developmental zone of their students and is thus able to support the students in ways that result in engagement and learning [51]. Consequently, a key condition for successful learning is a constant monitoring of the effects of the teacher’s actions. Another key condition is explicitly informing the student at the beginning of the lesson about the criteria for success and giving explicit feedback when these criteria are fulfilled [53]. Furthermore, teachers need to scaffold what Hattie [53] calls “an optimal proportion of surface and deep learning” and provide “appropriate levels of challenge”. Furthermore, he declares that the resulting learning is not only visible in test scores, but also has an impact through an increase in mastery, tolerance of coping with mistakes, degrees of collaboration, and motivation to learn more.

Hattie himself comments on some of the problems associated with the research reported in “visible learning;” for example, that he has given no attention to the effects of social background or context, because in his opinion these factors cannot be influenced in schools [51]. However, he attributes 50% of influences on learning to what the student brings to school and 10% of the influence is attributed to home environment contributions [54]. According to Snook, O’Neill, Clark, O’Neill, and Openshaw [55], there are two kinds of research on school effects. One kind of research is studying the relative contributions of social variables like SES and home resources to the contributions of school variables like curriculum, principal, teachers, and buildings. The other kind of research ignores social variables and asks only which school variables are important [55]. Hattie’s “visible learning” seems to belong to the latter group, although he does acknowledge that only 40% of the influences on learning comes from school variables. This lack of interest in SES factors becomes particularly problematic when considering the findings of a meta-analysis by Nye, Konstantopoulos, and Heedges [56] showing that the teacher factor was crucial for low-SES children, while for high SES-children, the quality of the teacher did not matter that much. Other critiques have dealt with problems of validity (there is a problem with clearly defining educational variables like achievement), Hattie’s lack of concern with the quality of studies included in the meta-analyses, publishing bias, oversimplifying as in the case of class size, overgeneralizing when averaging results across various subjects (reading, math, and science), and, finally basing conclusions on a collection of studies, including many that are several decades old [52,55,57]. Nevertheless, Terhart [52] concludes his critique of “visible learning” by stating that the book did not offer any surprises because most of the factors emerging from Hattie’s meta-meta-study were already known to be influential in learning, like teacher clarity, the teacher-student relationship, the quality of teaching, and teacher expectations, which are all concepts relevant to the theoretical basis of GC.

### 3.2. How the Theoretical Basis Is Applied in the Group Intervention

The GC intervention follows the basic principle of positive psychology by focusing on building strengths instead of fixing problems. By testing the boys’ competencies in math, reading, and writing before the camp, the teachers can start building on the students’ actual competencies from the first day of the camp and thus build extensive and authentic mastery experiences. The GC also includes the contextual positive psychology perspective by providing environments in which these young people can thrive and focus, thus teaching them to change their context to provide more vitality and meaning. Through a varied program and diverse activities, everybody is given a chance to excel. One example of acknowledgement and mastery-experiences is the assignment of the “Man of the Day” title. Every day, one of the boys is appointed “Man of the Day” for excelling in supporting his team or the entire group by contributing something special, like helping a teammate, volunteering for unpopular tasks, showing fairness, and so on. The chosen boy is called up in front of the entire group to receive the applause of all his teammates; he is given a diploma that is hung on the wall and handed to him at the graduation ceremony; and he also gets a selfie with the head of the camp and another staff member. This very simple acknowledgement of contributions to the group’s well-being is a very popular event in the day’s program and a much sought-after mastery experience among the boys. Many of these teenagers have learned little about their strengths in school and a lot about their shortcomings [4,5]. The fact that the camp is free of charge for the boys also adheres to the contextual positive psychology perspective, recognizing that not all participants bring the same resources to the table.

Introducing the boys to a wide variety of activities like basketball, swimming, table tennis, canoe paddling, ziplining, climbing, and games, while including them in social situations with a group of new peers, aims to build mastery experiences outside the classroom too. The activities are organized so as to give all the boys a chance to feel good about themselves, find their competencies and strengths, and experience belonging or, as Seligman [31] said about the focus of positive psychology, “identifying and nurturing their strongest qualities, what they own and are best at, and helping them find their niches in which they can live out these positive qualities”.

Nevertheless, GC is a learning camp, implying that teaching the subjects of reading, writing, and math is a main priority. Inspired by “visible learning”, the boys are met by several posters on the walls of the classroom; the posters display the character strengths and the rules of the camp. In math, one of the posters is a learning ladder where each step defines a certain competence: I can add, I can calculate percentages, I can do algebra, I can do equations, and so on. The boys get a sheet of tasks to work on and a list describing the requirements for success at each step of the learning ladder. When they think they have mastered one competence level on the ladder, through working on their task sheet and getting help from the teachers, they receive a quick test; when the test is passed, they go up to the poster and write their name on the current step of the learning ladder. This visualization of learning and success is followed by applause from the teachers and the other boys in their group. Thus, the boys constantly support each other’s academic progress, and the learning of the individual student becomes clear to the teacher and to the student through constant feedback to the teacher and the student about how the teaching and the learning are progressing. This can be understood as an implementation of Hattie’s [48] overall idea that teaching must be visible to the student and that the resulting learning must be visible to the teacher.

In reading classes, this need for visibility is also achieved by poster registration: Poster 1. Now I am reading…; Poster 2. So far, I have read…; and Poster 3. Small book reviews. Again, the point is to make it immediately visible to the individual boy what he has achieved and how far he has come in his reading efforts. This visualization has relevance because several of them have not read many books in their lives. Some boys finish reading their first book at the camp; therefore, every chapter matters. Books are chosen for their relevance in the boys’ lives and include themes boys are interested in like heroism, bullying, sports, love, friendship, discrimination, gaming, crime mysteries, growing up in minority groups, or fantasy novels.

There is hardly any group teaching using the blackboard with the whole group, just individual teacher–student contact and sometimes boys working together in a group and helping each other out. There are 3 teachers present in a group of about 15 boys. The teachers can always be called on, so that nobody will be stuck for long periods with problems they cannot solve. Such close individual follow-up and focus on the dialog between the teacher and the individual student also adheres to one of Hattie’s [51] core ideas: namely, the importance of the teacher–student relationship and the constant alertness to feedback about the progress of the learning process.

Finally, the GC has implemented Hattie’s ideas [51] regarding the importance of meta-strategies in learning and thus constantly works on building such competencies in “learning to learn”. The main strategy for leaning to learn is the focus on developing the six main character strengths from the Values in Action Youth Inventory (VIA-Y) [42], namely, interpersonal self-control, gratitude, social intelligence, zest, curiosity, academic self-control, and grit. The inclusion of these strengths in the everyday life at the camp is accomplished in various ways. For example, every day at camp, one of the six character strengths is focused on. The teachers wear T-shirts promoting the strength of the day, and the rooms at camp are decorated by posters promoting, describing, and clarifying the content of the strength of the day. In class, teachers actively demonstrate the strength of the day by pointing out to participants when this strength appears in the classroom and how it can be applied in their learning processes. For example, what does grit mean, how can grit be used, when are they showing grit, and how does the use of grit in learning situations contribute to their learning process? Teachers give feedback about how showing patience or self-control made them stay on task long enough to solve the problem, how self-control made them stay in the classroom when they wanted to give up and march out, and how, at the end of the day or the week, that results in their mastery of, for example, fractions or reading aloud to a small group.

However, the character strengths are not only meant to help participants develop meta-strategies for learning in the classroom. The GC program also emphasizes social competence and the ability to navigate life in general. Therefore, upon arrival, the boys are assigned to one of three teams named Alfa, Bravo, and Charlie. The teams stay together all through camp and continue as mentor groups. Participants go to class with their team, they do activities with their team, and they help and support each other in sports and various tasks to develop a solidarity and empathy with their team members under the motto “best together”. Many of the participants have reported during evaluation that these new acquaintances were among the most valuable elements of the camp. Character strengths are also applied in building team spirit and positive relationships by learning how gratitude promotes bonding and how optimism, grit, and zest will get you through team challenges. The social competence promoted is also about adapting to each other and everyday life, which means, for example, having table manners, taking turns, structuring time and tasks, concentrating by putting away your mobile phone, taking care of yourself by eating healthy, and getting enough sleep. Consequently, the participants learn how various character strengths influence and stimulate each other so that improving their relationships evokes individual mastery experiences and how building character strengths like optimism, self-control, and curiosity can help them develop more giving relationships.

After the camp, the boys meet up with their teammates and teachers from the camp twice a month for one and a half years at what are called mentoring centres. The purpose of these meetings is to keep track of the boys’ academic and social development. The boys will get help with their homework, they can share problems concerning their classroom behaviour or their academic challenges, and they can plan strategies for coping with school problems and social relations. However, the social aspect of meeting up with their teammates from the camp, eating pizza, playing table tennis or basketball, and having fun together is always an important element in these meetings.

## 4. Discussion

New and efficient ways of helping demotivated secondary school students complete high school are in high demand. Providing interventions that can prevent high school dropout and the risk of subsequent marginalization is a prioritized task in EU2020 strategy [58]. In Norway, one of the new additions in this line of work is the GC intervention. Above, we have analysed and described core elements in the theoretical basis on which the GC intervention is built and furthermore described how these theoretical perspectives are integrated into the teaching, organization, and social interaction at the camp and mentoring centres.

A main finding in this study is that the central theoretical basis of GC has been positive psychology’s focus on human strengths and positive attributes or, put in different words, what makes life worth living [29,30]. Second, we find that GC is also clearly influenced by the positive education approach in combining positive psychology with best practice teaching to promote optimal development and well-being in the school setting [36]. However, in their effort to identify and nurture the boys’ strongest qualities, there is a risk, as pointed out by the context-focused positive psychology (CPP) approach, to underrate the relevance of contextual factors and to undermine the relevance of negative emotions. Consequentially, it has been pivotal to include elements of the Discoverer, Noticer, Advisor–Value model (DNA-V) in the theoretical basis of GC. This implies including a more explicit purpose of learning to cope with adversities and challenges while adhering to one’s basic values. Third, character strengths may go a long way in this regard, but adding a deeper understanding of how these strengths are realized using the DNA-V model has proven to be a helpful expansion of the theoretical basis of GC. Fourth, according to Hattie’s [48] comprehensive review, there is substantial support for the idea that the monitoring of and the reception of feedback in the learning process is pivotal to the learner’s motivation and engagement and to the quality of the teaching. Thus, the concept of visible learning has been helpful when trying to remotivate wounded learner identities [11].

In addition to these four theoretical pillars, there are other theoretical frames that might have been included in our overview of theories that have influenced the shaping of the GC model. For example, the social emotional learning (SEL) framework, with its focus on learning how to integrate your feelings, thinking, and behaviour in successful realization of important life tasks [59,60], may have influenced the program. Furthermore, cognitive learning theory, with its meta-cognition framework [61], might also have influenced the emphasis that GC places on implementing character strengths to promote learning. However, these potential theoretical influences have not explicitly been acknowledged as part of the GC’s theoretical basis (i.e., in the data that we examined).

We believe that the purpose of the GC program must be taken into consideration when discussing the programme’s theoretical basis. In addition to helping individual students achieving their goals—an important contribution of GC as we see it—is to create a laboratory for researching teaching and learning outside the school system, because changing large systems is challenging. Testing well-founded ideas by means of an intensive learning camp might be an efficient way of studying and leaning about what to change in school teaching and how. In the context of such an intensive learning camp, it may be impossible and even unnecessary to simultaneously explore all relevant theoretical frameworks and concepts involved in promoting learning. Identifying a few central theoretical pillars and then gradually including other influential factors based on experiences with the camps might be a sensible way to progress. In this manner, the GC intervention might lead to insights that can be adopted by other interventions or even by the general school system.

Considering future research, it is crucial to understand what the intervention is and how it is expected to work. Greenhalgh et al. [62] point to the fact that many researchers have recognized the importance of a theory-driven approach. Failing to make this kind of clarification, will, they claim, complicate the process of selecting the criteria on which to base the judgement of its potential effects. To choose the best criteria for evaluating the effects of the GC intervention, it is critical to know which mechanisms are involved and in what contexts the effects are achieved. If interventions do not result in the expected outcomes, it is important to have sufficient knowledge of how and when the intervention should work in order to disentangle why these outcomes were not achieved.

So far, the evaluations made after each camp and a study investigating the Danish version of this intervention [22] give some hope as to the effect of this type of intervention. However, more research is needed to broaden our understanding of how intervention programs in general can reduce the risk of school dropout and of the short- and long-term effects of the GC intervention and the experiences of its participants and stakeholders. Accordingly, our study of the theoretical underpinnings of the GC program will be followed by an empirical study. We have, as a next step, set out to interview participants in the 2022 summer camp in order to explore their perceptions regarding the program’s effects on learning, school engagement, and well-being.

## 5. Conclusions

In this study, we have examined the theoretical basis of the GC dropout-prevention program. We have identified and discussed the four main theoretical pillars on which the program builds: positive psychology, positive education, character strengths, and visible learning. In addition, we have demonstrated how these theories are applied in practice in the group intervention. More research is needed to examine the effects of the program and to form the basis of future adjustments and improvements to the program.

## Data Availability

Not applicable.

## References

[1] Lillejord S., Halvorsrud K., Ruud E., Morgan K., Freyr T., Fischer-Griffiths P., Eikeland O.J., Hauge T.E., Homme A.D., Manger T. (2015). Frafall i Videregående Opplæring: En Systematisk Kunnskapsoversikt [Student Dropout in Upper Secondary Education–A Systematic Review].

[2] Rumberger R.W., Lim S.A. (2008). Why Students Drop Out of School: A Review of 25 Years of Research.

[3] De Ridder K.A., Pape K., Cuypers K., Johnsen R., Holmen T.L., Westin S., Bjørngaard J.H. (2013). Upper secondary school dropout and long-term sickness and disability in young adulthood: A prospective propensity score stratified cohort study (the Young-HUNT study). BMC Public Health.

[4] Ramsdal G., Gjærum R.G., Wynn R. (2013). Dropout and early unemployment. Int. J. Educ. Res..

[5] Ramsdal G.H., Bergvik S., Wynn R. (2018). Long-term dropout from school and work and mental health in young adults in Norway: A qualitative interview-based study. Cogent Psychol..

[6] Ramsdal G., Bergvik S., Wynn R. (2015). Parent–child attachment, academic performance and the process of high-school dropout: A narrative review. Attach. Hum. Dev..

[7] Ramsdal G., Wynn R. (2014). Parental involvement and drop-out from high school. Eur. Psychiatry.

[8] Rumberger R.W., Lamb S.P. (2003). The early employment and further education experiences of high school dropouts: A comparative study of the United States and Australia. Econ. Educ. Rev..

[9] Ramsdal G., Wynn R. (2020). Re-enrolling young people who had dropped out of high school. A follow up study describing experiences with a public intervention program. Eur. Psychiatry.

[10] Ramsdal G.H., Wynn R. (2021). How young people who had dropped out of high school experienced their re-enrolment processes. Int. J. Educ. Res..

[11] Ramsdal G.H., Wynn R. (2022). Attachment and school completion: Understanding young people who have dropped out of high school and important factors in their re-enrollment. Int. J. Environ. Res. Public Health.

[12] Ramsdal G., Wynn R. (2022). Use of technology during the Covid-19 pandemic in a program for re-enrolling high school dropouts. Int. J. Integr. Care.

[13] Markussen E., Daus S., Bedre S. (2020). 14År Etter Avsluttet Ungdomsskole–Slik Gikk Det [14 Years after Completed Middle School–This Is How It Went]. https://www.utdanningsnytt.no/fagartikkel-videregaende-opplaering/14-ar-etter-avsluttet-ungdomsskole--slik-gikk-det/263640.

[14] Markussen E. (2019). Karakterene Fra Tiende Betyr (nesten) alt. Om Gjennomføring, Kompetanseoppnåelse og Slutting i Videregående Opplæring Gjennom Fire år for Unge Som Gikk ut av Ungdomsskolen i BERGEN Våren 2014 [The Grades from the Final Year in Middle School Mean (almost) Everything. About Completion, Competency and Drop Out from High School During Four Years for Youth that Completed Middle School in Bergen Spring 2014]. NIFU Rapport 20.

[15] Alivernini F., Cavicchiolo E., Manganelli S., Chirico A., Lucidi F. (2019). Support for autonomy at school predicts immigrant adolescents’ psychological well-being. J. Immigr. Minor. Health.

[16] Falch T., Borge L., Lujala P., Nyhus O.H., Strøm B. (2010). Årsaker Til Og Konsekvenserav Manglende Fullføring Av Videregående Opplæring [Causes and Consequences of Drop Out from High School].

[17] Øia T. (2011). Ungdomsskoleelever. Motivasjon, Mestring og resultater (Middle School Students. Motivation, Coping and Results). NOVA rapport 9.

[18] Grønli K.S. (2014). Mer Videregående Gir Mindre Kriminalitet [More High School Results in Less Crime]. Forskning.no.https://forskning.no/norges-forskningsrad-arbeid-skole-og-utdanning/mer-videregaende-gir-mindre-kriminalitet/563818.

[19] Bragstad T. (2018). Vekst i uføretrygding blant unge [Increase in disability benefits in youth]. Arb. Og Velferd.

[20] NOKUT (2017). General Information about Education in Norway, 4/10/2917. https://www.nokut.no/en/norwegian-education/general-information-about-education-in-norway/.

[21] Markussen E., Frøseth M.W., Sandberg N. (2011). Reaching for the unreachable: Identifying factors predicting early school leaving and non-completion in Norwegian upper secondary education. Scand J. Educ. Res..

[22] Rosholm M., Nielsen S.A., Hvidman C. (2020). Effektevaluering av Tre Intensive Læringsforløb [Effect Evaluation of Three Intensive Learning Courses].

[23] Roald K. Evaluering av Guttas Campus Kull 2021 Oslo (Evaulation of Guttas Campus Class of 2021 Oslo). http://www.guttascampus.one/wp-content/uploads/2022/01/Evalueringsrapport-Oslo-2021.pdf.

[24] Andersen F.Ø., Nissen P., Poulsen L. (2016). Inclusion of marginalized boys: A survey of a summer school using positive psychology interventions. J. Educ. Issues.

[25] Tronsmo P. Guttas Campus. *Kontekst Online*
**2019**, *3*. https://www.nubu.no/pdf-generator.php?url=https%3A%2F%2Fwww.nubu.no%2F&aid=2983&token=7146c8348d8c91d22b643ef8d2455a55.

[26] Maslow A.H. (1981). Motivation and Personality.

[27] Seligman M.E. (2002). Authentic Happiness: Using the New Positive Psychology to Realize Your Potential for Lasting Fulfilment.

[28] Seligman M.E., Csikszentmihalyi M. (2014). Positive psychology: An introduction. Flow and the Foundations of Positive Psychology.

[29] Peterson C., Park N. (2003). Positive psychology as the even-handed positive psychologist views it. Psychol Inquiry.

[30] Diener E. (2009). The Science of Well-Being.

[31] Seligman M.E. (2002). Positive psychology, positive prevention, and positive therapy. Handbook of Positive Psychology.

[32] Galli F., Giancamilli F., Palombi T., Vitale J.A., Borghi S., De Maria A., Cavicchiolo E., Diotaiuti P., La Torre A., Zelli A. (2022). Anxiety, motives, and intention for physical activity during the Italian COVID-19 lockdown: An observational longitudinal study. Int. J. Environ. Res. Public Health.

[33] Pettersen G., Rosenvinge J.H., Wynn R. (2011). Eating disorders and psychoeducation—Patients’ experiences of healing processes. Scand J. Caring Sci..

[34] Ciarrochi J., Atkins P.W., Hayes L.L., Sahdra B.K., Parker P. (2016). Contextual positive psychology: Policy recommendations for implementing positive psychology into schools. Front. Psychol..

[35] Seligman M.E., Ernst R.M., Gillham J., Reivich K., Linkins M. (2009). Positive education: Positive psychology and classroom interventions. Oxford Rev. Educ..

[36] Norrish J.M., Williams P., O’Connor M., Robinson J. (2013). An applied framework for positive education. Int. J. Wellbeing.

[37] Seligman M.E. (2012). Flourish: A Visionary New Understanding of Happiness and Well-Being.

[38] Jones E.E., Harris V.A. (1967). The attribution of attitudes. J. Exp. Soc. Psychol..

[39] Chawla N., Ostafin B. (2007). Experiential avoidance as a functional dimensional approach to psychopathology: An empirical review. J. Clin. Psychol..

[40] Cohen G.L., Sherman D.K. (2014). The psychology of change: Self-affirmation and social psychological intervention. Ann. Rev. Psychol..

[41] Peterson C., Seligman M.E. (2004). Character Strzengths and Virtues: A Handbook and Classification (Vol. 1).

[42] Park N., Peterson C. (2006). Moral competence and character strengths among adolescents: The development and validation of the Values in Action Inventory of Strengths for Youth. J. Adolesc..

[43] Lavy S. (2020). A review of character strengths interventions in twenty-first-century schools: Their importance and how they can be fostered. Appl. Res. Qual. Life.

[44] Park D., Tsukayama E., Goodwin G.P., Patrick S., Duckworth A.L. (2017). A tripartite taxonomy of character: Evidence for intrapersonal, interpersonal, and intellectual competencies in children. Contemp. Educ. Psychol..

[45] Tough P. (2012). How Children Succeed: Grit, Curiosity, and the Hidden Power of Character.

[46] Lickona T., Davidson M. (2005). Smart & Good High Schools: Integrating Excellence and Ethics for Success in School, Work, and Beyond.

[47] Baehr J. (2013). Educating for intellectual virtues: From theory to practice. J. Philosoph. Educ..

[48] Ritchhart R. (2008). Intellectual Character: What It Is, Why It Matters and How to Get It.

[49] Baehr J. (2016). Intellectual Virtues and Education.

[50] Pellegrino J.W., Hilton M.L. (2012). Education for Life and Work: Developing Transferable Knowledge and Skills in the 21st Century.

[51] Hattie J. (2008). Visible Learning: A Synthesis of over 800 Meta-Analyses Relating to Achievement.

[52] Terhart E. (2011). Has John Hattie really found the holy grail of research on teaching? An extended review of Visible Learning. J. Curric. Stud..

[53] Hattie J. (2015). The applicability of visible learning to higher education. Scholarsh. Teach Learn. Psychol..

[54] Hattie J.A.C. (2003). Teachers Make a Difference: What Is the Research Evidence? Paper Presented at the BUILDING Teacher Quality: What Does the Research Tell Us ACER Research Conference, Melbourne, Australia. http://research.acer.edu.au/research_conference_2003/4/.

[55] Snook I., O’Neill J., Clark J., O’Neill A.M., Openshaw R. (2009). Invisible learnings? A commentary on John Hattie’s book-‘Visible learning: A synthesis of Over 800 meta-analyses relating to achievement’. N. Z. J. Educ. Stud..

[56] Nye B., Konstantopoulos S., Hedges L.V. (2004). How large are teacher effects?. Educ. Eval. Policy Anal..

[57] Sjøberg S. (2012). ‘Visible Learning’: Ny giv for norsk skole. Utdanning.

[58] Bäckman O., Jakobsen V., Lorentzen T., Österbacka E., Dahl E. (2015). Early school leaving in Scandinavia: Extent and labour market effects. J. Eur. Soc. Policy.

[59] Newman J., Dusenbury L. (2015). Social and emotional learning (SEL): A framework for academic, social, and emotional success. Prevention Science in School Settings.

[60] Zins J.E., Payton J.W., Weissberg R.P., O’Brien M.U. (2007). Social and emotional learning for successful school performance. The Science of Emotional Intelligence: Knowns and Unknowns.

[61] Nelson T.O. (1992). Metacognition: Core Readings.

[62] Greenhalgh J., Long A.F., Flynn R. (2005). The use of patient reported outcome measures in routine clinical practice: Lack of impact or lack of theory?. Soc. Sci. Med..

